# Health Research Funding in Mexico: The Need for a Long-Term Agenda

**DOI:** 10.1371/journal.pone.0051195

**Published:** 2012-12-10

**Authors:** Eduardo Martínez-Martínez, María Luisa Zaragoza, Elmer Solano, Brenda Figueroa, Patricia Zúñiga, Juan P. Laclette

**Affiliations:** 1 Coordinación de Estadística y Proyectos, Foro Consultivo Científico y Tecnológico A. C., Colonia del Valle, Benito Juárez, Distrito Federal, México; 2 Departamento de Inmunología, Instituto de Investigaciones Biomédicas, Universidad Nacional Autónoma de México, Coyoacán, Distrito Federal, México; Vanderbilt University, United States of America

## Abstract

**Background:**

The legal framework and funding mechanisms of the national health research system were recently reformed in Mexico. A study of the resource allocation for health research is still missing. We identified the health research areas funded by the National Council on Science and Technology (CONACYT) and examined whether research funding has been aligned to national health problems.

**Methods and Findings:**

We collected the information to create a database of research grant projects supported through the three main Sectoral Funds managed by CONACYT between 2003 and 2010. The health-related projects were identified and classified according to their methodological approach and research objective. A correlation analysis was carried out to evaluate the association between disease-specific funding and two indicators of disease burden. From 2003 to 2010, research grant funding increased by 32% at a compound annual growth rate of 3.5%. By research objective, the budget fluctuated annually resulting in modest increments or even decrements during the period under analysis. The basic science category received the largest share of funding (29%) while the less funded category was violence and accidents (1.4%). The number of deaths (ρ = 0.51; P<0.001) and disability-adjusted life years (DALYs; ρ = 0.33; P = 0.004) were weakly correlated with the funding for health research. Considering the two indicators, poisonings and infectious and parasitic diseases were among the most overfunded conditions. In contrast, congenital anomalies, road traffic accidents, cerebrovascular disease, and chronic obstructive pulmonary disease were the most underfunded conditions.

**Conclusions:**

Although the health research funding has grown since the creation of CONACYT sectoral funds, the financial effort is still low in comparison to other Latin American countries with similar development. Furthermore, the great diversity of the funded topics compromises the efficacy of the investment. Better mechanisms of research priority-setting are required to adjust the research portfolio to the new health panorama of Mexican population.

## Introduction

During the 20^th^ century, the health conditions of the world population improved at an accelerated pace. Medical advances mostly originated in developed countries have spread worldwide. The use of antibiotics, vaccines, new drugs, and family planning has benefited the population of low-and middle-income countries. This fact is clearly reflected in the average life expectancy which in some of these countries approaches today the figures already achieved in developed countries. Despite these great advancements, there are still considerable health disparities between and within countries [1]. Moreover, regions like Latin America are now facing an epidemiological transition characterized by new health challenges parallel to the declining, but still active old problems. Long-term local and regional strategies are required to promote an effective performance of national health systems.

It is generally accepted that the efficiency of a national health system is based on its capability to generate and use high-quality scientific knowledge, as much as on the adequate financing and governance. Health research is not only important to a better understanding of diseases, but also is crucial for the evaluation of interventions and generation of new diagnostics, prophylactic tools, and treatments. In the past few years, Latin American countries have focused on the development of a National Health Research System [2], [3]. In the case of Mexico, the health system was reformed in 2003 to provide universal health-care by introducing a series of financial innovations and a redefinition of the stewardship of the Ministry of Health [4], [5]. This reform is having a positive impact on the health service access, as well as on reducing catastrophic health expenditures by individuals, thus reducing social inequality [4], [6], [7]. A key aspect of this and other reforms that have shaped the Mexican health system since the late 1970s, is that health policy is based on reliable indicators derived from large-scale national surveys linked to organizational and econometric analyses [5], [8]. This evidence-based health intervention has been possible in part by a concomitant growing and strengthening of research institutions in Mexico.

The health research sector was among the first scientific sectors in Mexico operating under institutional framework with modern scientific practices [9], [10]. Research groups originated during the 1940's were placed in high specialty hospitals. New groups, mainly dedicated to biomedical research, began to operate within universities and other public research centers [9]. Although the Mexican scientific production on health research has steadily increased over the years, the new research topics are not always linked to the health needs of the country [11], [12], [13]. To some extent, this is the result of a lack in the definition of the research agenda by health authorities leaving researchers to the pursuit of individual goals. The identification of large, ambitious projects oriented to health needs has been hampered by the lack of a financial structure ensuring medium and long-term funding. On the other hand, collaboration between researchers and decision makers on these projects has faced a tradition of individualistic work [14]. Previous analyses have suggested that most of research activity is not related to government health policies or to pharmaceutical industry needs [12], [15].

Under the current organization in Mexico, the Ministry of Health has the stewardship to directly orient and regulate the research activity of its own service providers, which include 13 National Institutes of Health and many other public hospitals. In addition, the Law for Science and Technology approved in 2002, dictated the creation of trust funds, formed by equal contributions from the National Council on Science and Technology (CONACYT) and other public entities. The Sectoral Fund for Research in Health and Social Security (FOSISS), established between CONACYT and the Ministry of Health, the Mexican Institute of Social Security (IMSS) and the Institute for Social Security and Services for State Workers (ISSSTE), was created to assist the national health priorities and to foster technological development. Additional investments in health-related projects, especially on biomedical disciplines, can also come from the partnership between CONACYT and the Ministry of Education, represented by the Sectoral Fund for Basic Research (FOSIB); as well as, from the partnership between the Governments of each of the 32 State and CONACYT, represented by the Mixed Funds (FOMIX). Despite the importance of a continuous surveillance on the allocation of public resources to steer the system towards socially agreed goals, a comprehensive analysis of health research resources is still missing.

Here we report for the first time a detailed estimate of how the public funding has been allocated to health research and its alignment with the needs of the Mexican health system. We determined the areas of health research that have been supported through the analysis of individual projects that were funded between 2003 and 2010. Our results showed that the research budget has supported a large number of small and diverse projects, indicating an unclear priority definition. Moreover, there was a weak correlation between the subjects favored by funding and the measures of disease burden. Our study is comparable to parallel efforts to track the flow of financial resources invested on health research reported for other countries [16], [17], [18], [19], [20].

## Methods

To examine the flow of public resources towards health research, we constructed a database including all projects financed by CONACYT through specific funding programs. CONACYT is the federal council in charge of defining and implementing the general policies for the functioning of the national system of science technology and innovation in Mexico. Since 2002, the allocation of the resources for research projects is made through the creation of trust funds in coordination with ministries, state governments or other entities of the public administration. The declared purpose of these funds is to support research projects tackling defined priorities in each sector or region of the country. By the end of 2011, CONACYT had integrated 55funds with national public entities and one fund for international cooperation. Information collected for this study included the fund with the Ministry of Health (FOSISS), the fund with the Ministry of Education (FOSIB) and the 34 funds with state and municipal governments (FOMIX). Most of the health-related research projects were included in these funds. Altogether, these funds involved 81% of the research grants funded in the period 2003–2010. An important aspect was the availability of annual information for each fund during the period of study.

The CONACYT Funds are managed by a technical and administrative committee composed of representatives from CONACYT, the corresponding public entity of each fund, and a number of specialists from the academic or private sectors. This committee is supposed to identify the needs and problems within a sector before establishing the goals and requirements for the submission of research proposals. An evaluation team aids the committee to select the proposals that are aligned with the sector demands. This process for application and evaluation of proposals is basically the same for FOSSIS, FOSIB and FOMIX. FOSSIS has also included a pre-selection stage based on evaluation of pertinence of the proposals through the revision of an executive summary. Fundable items include current expenses (purchases of materials, travel and accommodation expenses, student and postdoctoral fellowships and other professional fees) and capital expenses (purchases of equipment, computers and software, expenses for building or remodeling experimental facilities, etc.). Between 2003 and 2011, the financial contribution of CONACYT to the funds was about 57% for FOSIB and FOSISS and an average of 56% for FOMIX.

Data were captured on an Excel spreadsheet containing the following columns: project identification number, year, project title, principal investigator, institution, state, and total amount approved. All projects included in FOSISS were considered for the analysis. Because FOMIX and FOSIB support projects in all areas of knowledge, the next step was the identification of health research projects. Our inclusion criterion for a health-related project was the identification of a sector of activity or application for health [17]. Hence, selected projects involved medical and natural sciences, as well as research on health determinants, health economy, sociological studies and development of new applications for improving the health of groups and individuals. It is worth mentioning that some projects involved areas of knowledge such as humanities and engineering.

The matrix for health research projects followed the 27 categories employed in a recent analysis of the financial flows for health research in 5 Latin American countries [18], [19]. Each project was classified according to its title into one of three possible research methodologies: biomedical, clinical or public health research and then crossed with nine thematic objectives [19]. Thematic objectives were grouped in three main groups: health determinants (1. social, economic and cultural factors), health conditions (2. communicable diseases and maternal and perinatal conditions; 3. noncommunicable diseases and addictions; 4. nutrition and the environment; 5. violence and accidents), and health actions or interventions (6. health policy, systems and services, 7. technological research and development, 8. basic research, and 9. traditional medicine). If a research project included more than one thematic objective, the total funds were divided equally among the objectives covered by the project [18]. In order to avoid misclassifications of projects, we also consulted the laboratory website of the principal investigator and his published work. Within FOMIX, we identified 49 projects that were considered by CONACYT as health-related. However, we were unable to classify them within either a research methodology or an objective category. These projects were directed to infrastructure investment, support of graduate programs, publication fees, and meetings organization. In our analysis we did not evaluate the pertinence of the results with the original proposal based on a follow up of the final report, as this information was not available. Other figures related with public global resources directed to health and R&D, including CONACYT, were based on budget reports from the Ministry of Finance [21].

We also analyzed the correlation of health research fund allocations with parameters of the burden of disease in Mexico as described previously [16], [22]. For this purpose, we searched the names of specific diseases or medical terms associated with certain diseases within project titles. Diseases were classified using the International Classification of Diseases, 10^th^ revision (ICD-10 code) [23]. In case of projects involving several diseases, funding was only weighted when the diseases corresponded to different groups of de ICD-10 code. Estimates of mortality and disability-adjusted life-years lost (DALY) were obtained from the 2004 update of the Global Burden of Disease project of the World Health Organization [24]. Correlation coefficients were determined with the non-parametric Spearmans’s rho test. The predicted values of CONACYT funding were obtained by simple linear regression for log-transformed data, with funding as the dependent variable and a measure of disease as the independent variable. The predicted values were compared with the actual funding, to assess which diseases were overfunded or underfunded. Regression models were performed with the Stata 12 software (College Station, Texas, USA).

For comparative purposes, the original cash amounts in current Mexican pesos were converted to US dollars, adjusted for purchasing power parity (US$ PPP). To carry out this conversion, we used the PPP conversion factor (GDP) to market exchange rate ratio, from the World Development Indicators of the World Bank. Cash amounts used in this report correspond to the total amounts approved per fund, per year. The analysis was based on awarded funds for research grants. CONACYT resources devoted to cover fellowships (salary incentives) for the National System of Researchers, as well as funding for the Postgraduate Studies Scholarships Program were not included in this analysis.

## Results

The context in which CONACYT funds operated during the period 2003–2010, within the federal budget for health and for research and development, is presented in Table 1. Total federal budget on health increased from $ 20.4 billion in 2003 to $ 39 billion in 2010 at a compound annual growth rate of 8.4%. The spending for health included some resources for research and development that accounted for $ 2.4 billion in the seven-year period. These resources also increased at a compound annual rate of 3.1%. Similarly, the total federal resources directed to support research and development (R&D) increased at a growth rate of 3.3%. In contrast, the share R&D within health decreased about 40% compared to the total federal budget and 49% compared to the budget for health and social security.

**Table 1:** Federal Government budget for Health, R&D, and Health R&D*.

	2000	2003	2004	2005	2007	2008	2009	2010
	Millions of US$ PPP							
**Total Federal Budget** 1	**194,660**	**223,707**	**228,731**	**255,350**	**308,518**	**343,556**	**395,057**	**398,908**
**Budget for Health and Social Security** 2	**17,032**	**20,446**	**24,506**	**26,824**	**32,467**	**36,071**	**39,230**	**39,013**
% of total budget	8.7	9.1	10.7	10.5	10.5	10.5	9.9	9.8
**Budget for R&D** 3	**3,758**	**4,300**	**3,874**	**4,401**	**4,434**	**5,226**	**5,647**	**5,577**
% of total budget	1.9	1.9	1.7	1.7	1.4	1.5	1.4	1.4
**Resources for R&D within Heath and Social Security**	**113**	**324**	**197**	**274**	**280**	**292**	**469**	**413**
% of total federal budget	0.058	0.145	0.086	0.107	0.091	0.085	0.119	0.104
% of Budget for health	0.66	1.59	0.80	1.02	0.86	0.81	1.19	1.06
% of resources for R&D	3.0	7.5	5.1	6.2	6.3	5.6	8.3	7.4

* Amounts in millions of US dollars adjusted for purchasing power parity (US$ PPP).

1 Refers to total net expenditure projected in the Expenditure Budget of the Federation for the corresponding fiscal year, Ministry of Finance (SHCP).

2 Includes the budget of Branch 12 Health, Mexican Institute of Social Security (IMSS) and Institute of Social Security for State Workers (ISSSTE) excluding the pension and retirement contributions. For 2009 and 2010 correspond to the Retirement and Pensions allocation of the branches GYN and GYR. From 2002 to 2008 corresponds to the item “old age” in the branches GYN and GYR. Data for 2000 and 2001 were taken from the Branch 19 “Contributions to Social Security” specified for each entity.

3 Includes the resources for science and technology allocated to National Council on Science and Technology (CONACYT), Ministry of Education (SEP), Ministry of Energy (SENER), Ministry of Health (including IMSS and ISSSTE), Ministry of Agriculture, Livestock, Rural Development, Fisheries and Food (SAGARPA), Ministry of Economy (SE), Ministry of Environment and Natural Resources (SEMARNAT), Office of the Mexican Attorney-General (PGR), Ministry of Communications and Transport (SCT), Ministry of National Defense (SEDENA), Secretary of Navy (SEMAR) and Ministry of Tourism (SECTUR), including own resources.

4 Refers to the resources allocated to science and technology in the Branch 12 Health, IMSS and ISSSTE, including own resources.

Sources: Based on information of CONACYT, SHCP, Central Bank of Mexico (BANXICO), and World Bank.

**Figure 1:**
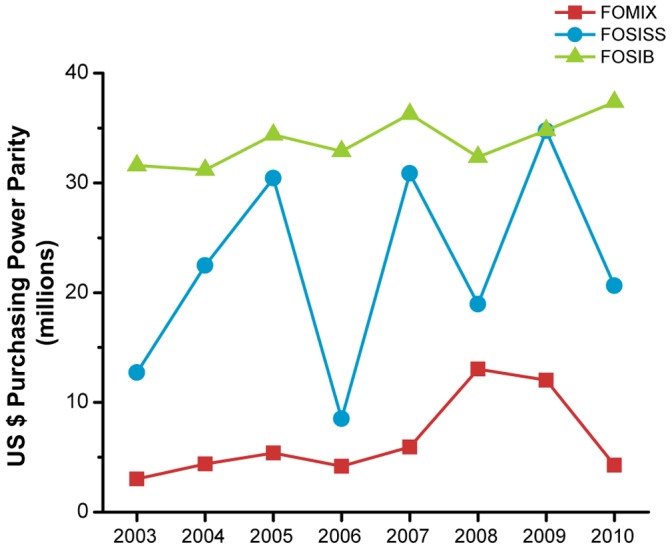
National Council on Science and Technology (CONACYT) investment on health research. The annual evolution of resources assigned to the Mixed Funds (FOMIX), Sectoral Fund for Research on Health and Social Security (FOSISS), and Sectoral Fund for Basic Research (FOSIB) is presented. The cash amounts are in US dollars adjusted for purchasing power parity (US$ PPP).

Considering CONACYT funds FOSSIS, FOSIB and FOMIX, 31.1% out of 10,603 projects in the database of the period under study were health-related (Table 2). The largest contributor was FOSIB with 55.5% of the health research projects, followed by FOSISS (28.2%) and FOMIX (16.4%). The average grant per project differed among the funds. The lowest average of funding was observed within FOMIX with an average of $ 94,967 per project whereas FOSIB and FOSISS received an average of $ 156,456 and $ 193,160, respectively. Although the resources in the three funds increased in real terms between 2003 and 2010, FOSISS showed an inconsistent behavior with strong drops in 2006 (71%), 2008 (38%), and 2010 (40%), in comparison with the former year (Fig. 1). Although FOMIX showed a growing tendency until 2008, it declined by 2010, returning to the funding level of 2006. Considering the three funds, the health research expenditure increased by 31.6% at a compound annual growth rate of 3.5% from 2003 to 2010. In this period, the average investment was of $ 5,771 per 10,000 inhabitants and the average coverage was of 3.79 projects per million inhabitants.

**Table 2:** General statistics of CONACYT^1^ funds, 2003–2010*.

	FOMIX^2^	FOSISS^3^	FOSIB^4^
**No. of projects funded**	3789	928	5886
**Total funding**	668.5	179.3	768.4
**Health research** **projects (%)**	539 (14)	928 (100)	1827 (31)
**Funding for health research projects (%)**	52.3 (7.8)	179.3 (100)	270.8 (35.2)

* Amounts in millions of US dollars adjusted for purchasing power parity.

1 CONACYT: National Council on Science and Technology.

2 FOMIX: Mixed Funds.

3 FOSISS: Sectoral Fund for Research in Health and Social Security.

4 FOSIB: Sectoral Fund for Basic Research.

**Figure 2:**
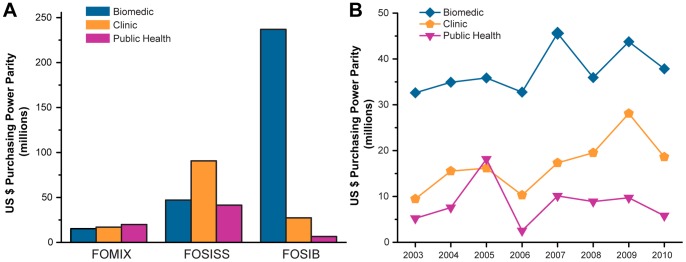
Evolution of the health research funding by research methodology. For each Sectoral Fund, the health-related projects were classified according to the methodology employed in biomedical research, clinical research, or public health research. The total amount awarded per methodology from 2003 to 2010 was estimated (A). Note that resources of Sectoral Fund for Basic Research (FOSIB) were mainly allocated to biomedical projects, whereas resources of Sectoral Fund for Research on Health and Social Security (FOSISS) were mostly directed to clinical projects. FOMIX: Mixed Funds.

Taking into account the methodological approaches of projects, FOSIB resources were primarily assigned to biomedical research (87.5%), although clinical (10.1%) and public health research (2.4%) were also supported (Fig. 2A). In contrast, FOSISS allocated half of its resources into clinical research and the other half was divided between biomedical and public health research. In FOMIX, the funding was distributed to approximately one third for each of the methodological areas considered. Overall, the biomedical area was the largest recipient of health research funding, accounting for $299 million in the analyzed period (Fig. 2B). Clinical research resources increased 99.9% from $9.5 million in 2003 to $18.9 million in 2010 at an annual growth rate of 8.8%. On the other side, funding for public health research peaked in 2005, but declined by 2006, and has maintained an intermediate funding since then.

**Figure 3:**
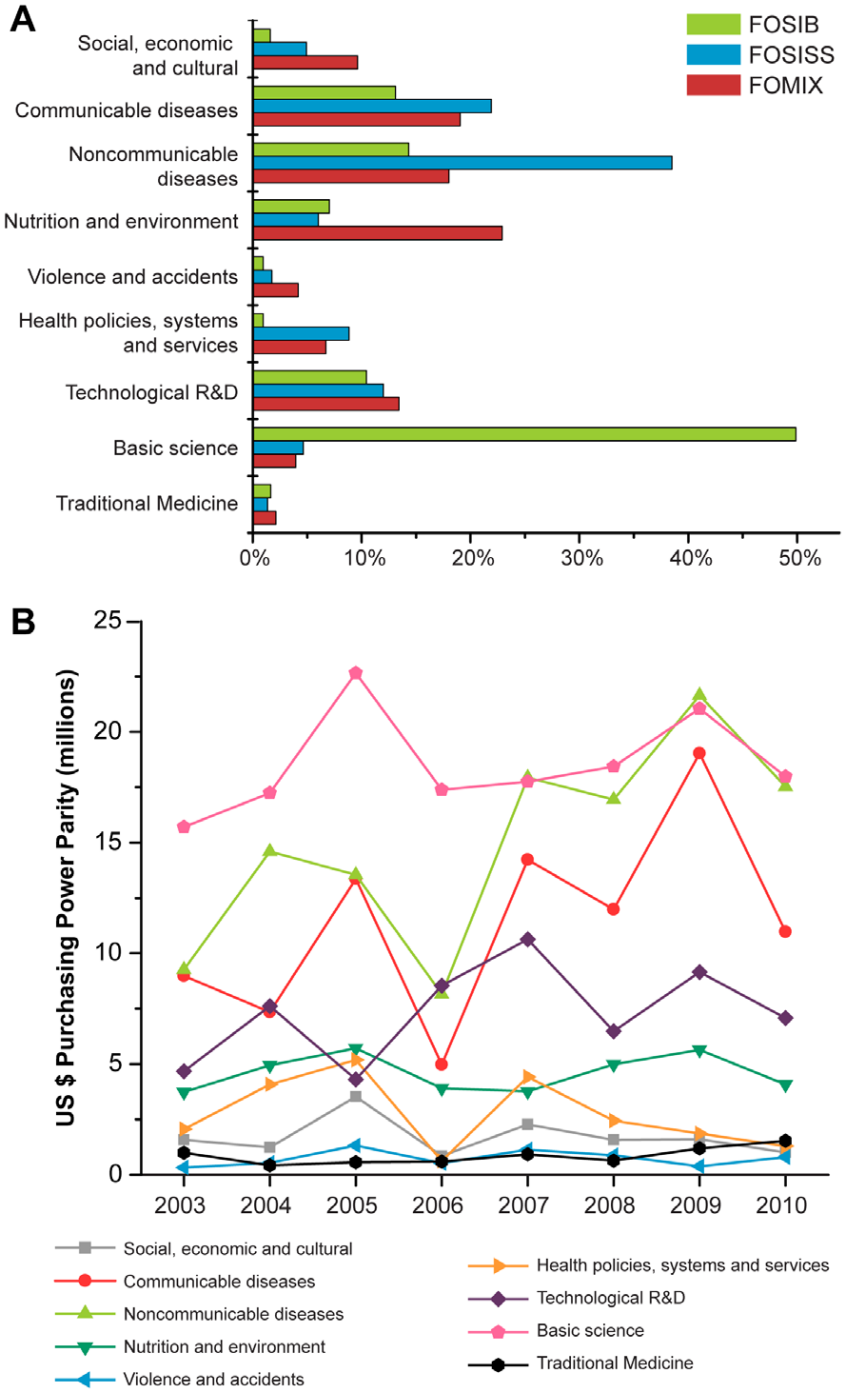
Project distribution by research objectives and annual amounts funded. A: The share for each research objective (in percent) within National Council on Science and Technology (CONACYT) funds. B: Budget fluctuations for each thematic objective over the period. Research projects were classified by thematic objective considering nine categories: health determinants (1.social, economic and cultural), health conditions (2. noncommunicable diseases; 3. communicable diseases; 4. nutrition and environment; 5. violence and accidents), and actions or interventions (6. research on health policy, systems and services; 7. technological R&D; 8. basic science; and 9. traditional medicine). FOSIB: Sectoral Fund for Basic Research; FOMIX: Mixed Funds; FOSISS: Sectoral Fund for Research in Health and Social Security.

By research objective (Fig. 3a), the largest portion of projects in FOSIB corresponded to basic science (50%) followed by noncommunicable diseases (14%) and communicable diseases (13%). In the case of FOSISS, a considerable support was provided to projects addressing noncommunicable diseases (38%), whereas communicable diseases and R&D issues accounted for 22% and 12%, respectively. Finally, in FOMIX the supported projects were related to nutrition and environment (23%), communicable diseases (19%), and noncommunicable diseases (18%). According to the annua1 evolution of the total funding allocated per research objective, basic research was the highest supported category, however, the thematic categories with the most dynamic behavior over the period were the noncommunicable diseases, communicable diseases and research and development (Fig. 3b). The last three categories accounted for 24%, 18% and 12% of the total funding for health research from 2003 to 2010 (Table 3). Nevertheless, basic science accounted for 29% of the total expenditures. In contrast, the thematic categories that showed a funding decline were the study of the social, economic and cultural determinants of health (−36%) and the research dealing with health policies, systems and services (−37%) (Fig. 3b). Finally, the category that received the lowest amount of funding over the period was the research on violence and accidents accounting for 1.2% of the total funding.

**Table 3:** Allocation of CONACYT^1^ (FOMIX^2^, FOSISS^3^, and FOSIB^4^) funds, by research methodology and objective (2003–2010)*.

	Research methodology			
Objective	Biomedical	Clinical	Public Health	Total
Social, economic and cultural	0.6	3.3	9.7	13.7
Communicable diseases	41.0	37.1	12.8	90.9
Noncommunicable diseases	37.5	69.0	13.0	119.6
Nutrition and environment	18.5	9.6	8.6	36.8
Violence and accidents	2.0	1.4	2.5	5.9
Health policies, systems and services	1.0	1.1	19.9	22.0
Technological R&D	53.9	3.1	1.5	58.5
Basic Research	138.3	9.8	0.1	148.2
Traditional Medicine	6.4	0.5	0	6.9
**Total**	**299.3**	**135.0**	**68.0**	**502.4**

* Amounts in millions of US dollars adjusted for purchasing power parity.

1 CONACYT: National Council on Science and Technology.

2 FOMIX: Mixed Funds.

3 FOSISS: Sectoral Fund for Research in Health and Social Security.

4 FOSIB: Sectoral Fund for Basic Research.

**Figure 4:**
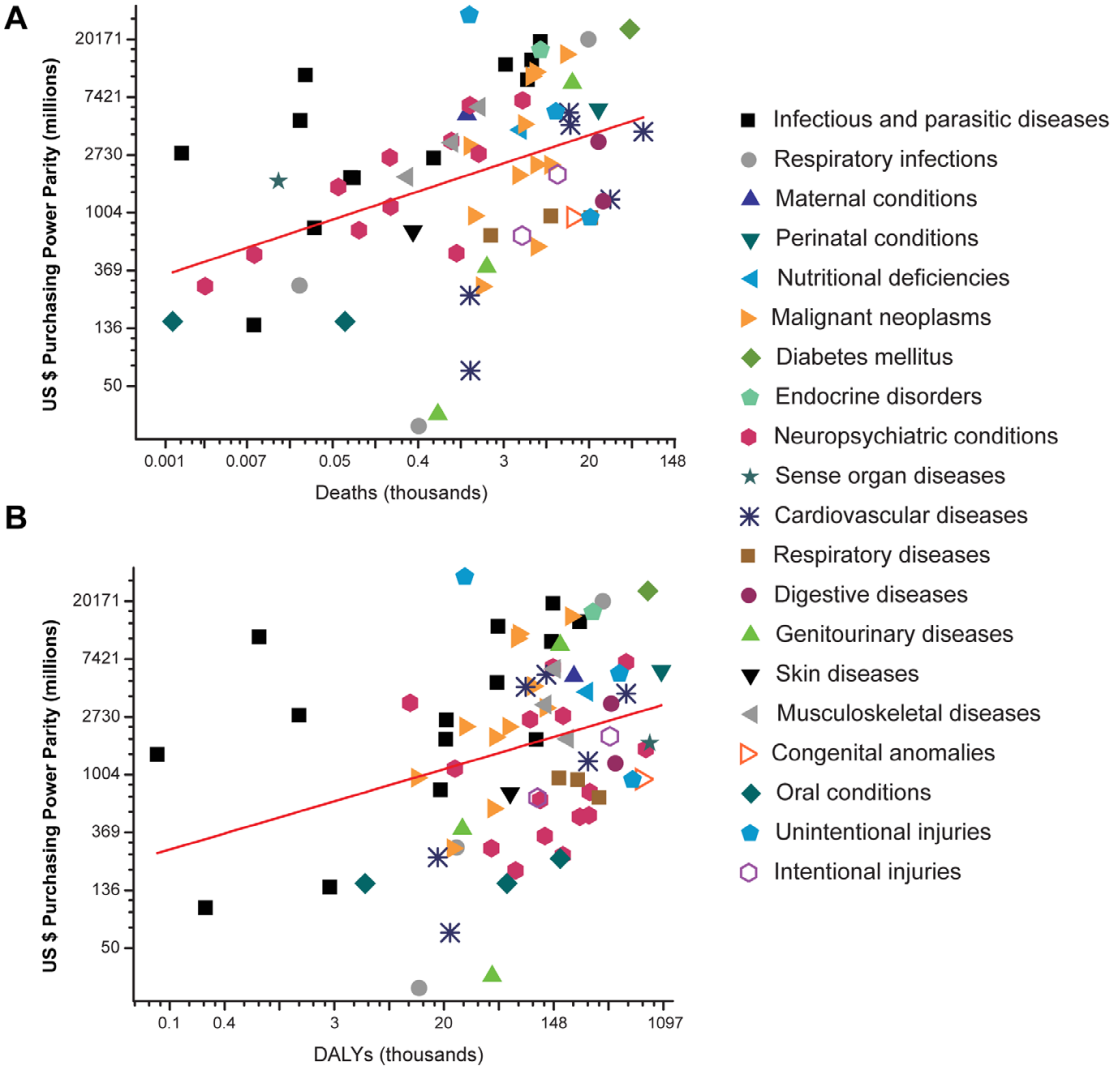
Relationship between CONACYT funding and disease burden. The scatter plots show the relationship between National Council on Science and Technology (CONACYT) funding and mortality (A) or disability-adjusted life years DALYs (B). The red line in each panel represents the predicted funding on the basis of the linear regression analysis of all data. Axes are on logarithmic scales.

Creation of CONACYT funds was supported by the concept of orienting the funding towards public needs. In the understanding that there is no a unique manner to establish research priorities, we examined whether the research projects supported by the CONACYT funds were aligned with measures of disease burden [25]. The analysis revealed that 2,190 (66%) out of 3,294 health projects were related to a specific disease. We also found that CONACYT funding was more closely associated with measures of mortality (ρ = 0.51, P<0.001) than with DALYs (ρ = 0.33, P = 0.004). Ten disease categories accounted for 53% of the resources allocated to specific diseases, but only diabetes mellitus (DALY rank = 3; Death rank = 2) and lower respiratory infections (Death rank = 9) were among the leading causes of disease burden in Mexico (Table S1). Through regression analysis of all points we defined the level between actual and desirable funding for each disease (Fig. 4). A negative value of the residual indicated that a disease was underfunded and vice versa, a positive value indicated that the disease was overfunded (Table 4). Five categories appeared underfunded when DALYs was used as predictor variable: congenital anomalies, road traffic accidents, alcohol use disorders, migraine and dental caries. On the other side, the five categories receiving more funding than recommendable were poisoning, diabetes mellitus, lower respiratory infections, infectious and parasitic diseases, and endocrine disorders. According to death measures, the five most overfunded categories remained the same, but the five most underfunded categories also included cerebrovascular disease, chronic obstructive pulmonary disease, and cirrhosis of the liver.

**Table 4:** Difference between actual and predicted funding on health research+.

Disease	DALY^1^ (rank)	Mortality(rank)	Disease	DALY (rank)	Mortality(rank)
	Thousands of US$ PPP*			Thousands of US$ PPP*	
**Congenital anomalies**	−2,089 (1)	−2,580 (5)	**Lymphomas, multiple myeloma**	486 (39)	−647 (23)
**Road traffic accidents**	−1,960 (2)	−2,948 (3)	**Childhood-cluster diseases**	736 (40)	865 (38)
**Alcohol use disorders**	−1,813 (3)	−1,309 (16)	**Epilepsy**	764 (41)	737 (36)
**Migraine**	−1,773 (4)	–	**Trachea, bronchus,** **lung cancers**	767 (42)	−776 (21)
**Dental caries**	−1,760 (5)	–	**Digestive diseases**	826 (43)	−646 (24)
**Asthma**	−1,755 (6)	−1,519 (14)	**Drug use disorders**	894 (44)	1,371 (43)
**Bipolar disorder**	−1,719 (7)	−85 (32)	**Prostate cancer**	1,079 (45)	−532 (25)
**Schizophrenia**	−1,574 (8)	−293 (28)	**Malaria**	1,165 (46)	–
**Unipolar depressive disorders**	−1,514 (9)	648 (35)	**Ischaemic heart disease**	1,290 (47)	−1,155 (18)
**Obsessive-compulsive disorder**	−1,499 (10)	–	**Gastrointestinal cancer**	1,334 (48)	1,241 (42)
**Cirrhosis of the liver**	−1,421 (11)	−2,947 (4)	**Hepatitis B&C**	1,474 (49)	1,008 (39)
**Sense organ diseases**	−1,409 (12)	1,080 (40)	**Rheumatoid arthritis**	1,519 (50)	1,607 (44)
**Insomnia (primary)**	−1,400 (13)	–	**Nutritional deficiencies**	1,926 (51)	1,611 (45)
**Benign prostatic hypertrophy**	−1,381 (14)	−1,593 (13)	**Leishmaniasis**	2,279 (52)	2,431 (50)
**Oral conditions**	−1,370 (15)	−804 (20)	**Parkinson disease**	2,536 (53)	1,713 (46)
**Chronic obstructive pulmonary disease**	−1,260 (16)	−2,960 (2)	**Perinatal conditions (h)**	2,707 (54)	1,991 (48)
**Panic disorder**	−1,154 (17)	–	**Leukaemia**	2,898 (55)	1,985 (47)
**Post-traumatic stress disorder**	−1,125 (18)	−150 (30)	**Hypertensive heart disease**	2,911 (56)	1,130 (41)
**Self-inflicted injuries**	−1,106 (19)	−1,952 (8)	**Other unintentional injuries**	3,065 (57)	2,583 (51)
**Inflammatory heart diseases (k)**	−1,076 (20)	−1,890 (9)	**Maternal conditions**	3,372 (58)	3,595 (52)
**Respiratory diseases**	−1,033 (21)	−2,142 (7)	**Chagas disease**	3,495 (59)	4,203 (54)
**Cerebrovascular disease**	−1,032 (22)	−3,074 (1)	**Cardiovascular diseases**	3,804 (60)	2,239 (49)
**Upper respiratory infections**	−951 (23)	−1,433 (15)	**Neuropsychiatric conditions**	4,223 (61)	4,379 (55)
**Respiratory infections**	−896 (24)	−455 (26)	**Musculoskeletal diseases**	4,314 (62)	4,197 (53)
**Ovary cancer**	−875 (25)	−1,805 (10)	**Alzheimer and other dementias**	4,517 (63)	4,492 (56)
**Liver cancer**	−860 (26)	−2,288 (6)	**Nephritis and nephrosis**	7,469 (64)	5,971 (57)
**Rheumatic heart disease**	−832 (27)	−1,709 (12)	**HIV/AIDS**	8,139 (65)	7,342 (58)
**Genitourinary diseases**	−822 (28)	−1,756 (11)	**Breast cancer**	9,005 (66)	7,844 (59)
**Skin diseases**	−815 (29)	−677 (22)	**Cervix uteri cancer**	9,878 (67)	8,666 (60)
**Violence**	−623 (30)	−1,264 (17)	**Dengue**	10,447 (68)	10,116 (61)
**Periodontal disease**	−588 (31)	−206 (29)	**Tuberculosis**	11,603 (69)	10,671 (62)
**Leprosy**	−477 (32)	−426 (27)	**Diarrhoeal diseases**	11,875 (70)	11,304 (63)
**Intestinal nematode infections**	−314 (33)	−32 (33)	**Malignant neoplasms**	13,450 (71)	12,186 (64)
**Onchocerciasis**	−229 (34)	–	**Endocrine disorders**	14,400 (72)	13,838 (65)
**Osteoarthritis**	−213 (35)	500 (34)	**Infectious and parasitic diseases**	17,516 (73)	16,530 (67)
**Multiple sclerosis**	−56 (36)	−129 (31)	**Lower respiratory infections**	17,612 (74)	16,264 (66)
**Melanoma and other skin cancers**	−10 (37)	−1,040 (19)	**Diabetes mellitus**	21,029 (75)	19,300 (68)
**STDs excluding HIV**	77 (38)	840 (37)	**Poisonings**	29,563 (76)	28,849 (69)

+ Negative values indicated that disease research was underfunded according to the predictor based on our linear regression model.

1 DALYs: Disability-adjusted life years.

* PPP: purchasing power parity.

## Discussion

The scientific systems of developing countries are commonly characterized by deficiencies in the alignment among the relevant actors and by a poor correlation between research activity and local needs. This circumstance often leads to an inefficient use of the limited public resources available for research and development. In this context, our analysis contributed to identify the funding trends in health research through the main source of public financing in Mexico. Although CONACYT funds have improved the support of science in Mexico, there are several aspects of their operation that need a reorientation to maximize the efficiency and social impact of the research investment. The fact that the Ministry of Health is only involved in one of the funds implies that over 60% of the resources for health research were allocated without an explicit link to the priorities defined by the federal head of the sector. As a consequence, the health research activity appears weakly associated with the health needs of the country. The lack of clear sectoral goals promotes the dispersion of the financial effort into a great variety of topics that are poorly associated to the burden of disease in Mexico.

As a result of the health reform, the budget for health increased steadily since 2003. Noteworthy, while the total budget for health almost doubled from 2003 to 2010, the resources for health R&D just increased 30% in the same period. Therefore, the share of health R&D as a percent of the total health budget decreased over the period. The average investment in health R&D during the analyzed period represented only 1% of the health budget, which corresponded to half of the World Health Organization recommendation [26]. Another sign of alert is the fact that the resources allocated for health policies, systems and services decreased over the period. It is worth mentioning that evidence-based decision making was one of the pillars supporting the construction of the current Mexican health system [5], [27]. Hence, it is advisable to preserve this input of information in order to make possible future adjustments of the health system, under a rational planning of interventions.

Since 2002, the legal framework strengthened the coordination between the actors and institutions of the Mexican health research system. The governance and management structure shares some characteristics with systems operating in other Latin American countries [2]. However, unlike several of the countries in the region, Mexico does not have a Ministry of Science and Technology. Therefore, the Ministry of Health, the Ministry of Education, and the State Governments negotiate separately their joint agendas with CONACYT. This structure can be considered a significant achievement in the establishment of mechanisms to coordinate the direction of the research activity within different sectors and regions. However, in the practice, some limitations can be identified. For example, FOSSIS can be considered a specialized fund, devoted to support clinical, biomedical and public health research; FOMIX also allocated resources based on an explicit list of predefined research priorities, whereas FOSIB awarded research grants based on a list of areas of knowledge. A major shortcoming of this organization has been the lack of consistency in the priorities or demands defined by each fund, as well as the absence of an explicit mechanism to avoid the duplication of efforts, or the concentration of resources in certain research topics or research groups that might not represent a priority area. Resources in FOSIB are allocated under more flexible premises than FOSSIS and FOMIX, because this fund was conceived to primarily support basic science. This is relevant, since FOSIB accounted for half of the financial resources on health research. As our analysis revealed, the diversity of research topics supported by this fund promoted that a substantial part of the scientific activity was not oriented to local or national problems [28].

Operation of CONACYT’s sectoral funds places Mexico within a limited group of Latin American countries, with the legal and financial instruments to focus the support on health research towards pertinent health problems [2], [17], [18]. As our figures on the federal budget for Health R&D only represent a partial amount of the total public investment on health R&D, we can only make a comparison of the growing trends of the recent data reported for Brazilian health R&D sector [17], [29]. Considering data of Brazil from 2000 to 2005, the federal budget for health R&D increased 52%, at annual compound growth rate of 7.28%. In the same period, Mexican federal budget for health R&D, as estimated in this work, increased 143% at an annual rate of 15.9% in the same period. However, during the period 2005 to 2010 the growth rate decreased, evidencing the lack of continuity on research policies. It is important to bear on mind that the aggregate figures considered in this work correspond to the federal budget predominantly managed by public federal health institutions. Thus, to get a more comprehensive view about the flow of financial resources, it is necessary to assess in future studies, the participation of state governments, universities, as well as the private sector and international funding. Despite this limitation, our analyses allowed to make some inferences about the final destiny of the resources. Recently, it was reported that salaries accounted for the majority of the expenses on research within the National Institutes of Health in Mexico (range: 48% to 84%; average: 70%), which are the main recipients of federal budget for health R&D [25]. Interestingly, the percentage that represents the amount of health research grants of the three CONACYT funds in relation to the federal budget for health R&D has decreased from 2004 to 2010 (data not shown). Thus, it is possible that salaries and administrative costs are now taking a bigger part of the resources previously directed to research activities. In fact, this could be a general trend in the country, as the number of researchers registered in the National Researchers System is growing rapidly (7,982 researchers in 2002 to 17,568 researchers in 2011). Thus, the rate of incorporation of new researchers is growing faster than the available resources to fund research grants. If available funds for grants do not parallel the growth in the number of researchers, the development of new generations of health researchers is at stake [30].

The values and interests of the diverse groups composing modern societies often lead to tensions regarding the decision making about public issues. The research priority-setting process is not exempt of this complexity. However, in developed countries the mechanisms of priority setting and evaluation of research impact are regularly updated [16], [31], [32], [33]. In contrast, the national research systems in low and middle income countries rarely have explicit mechanisms for priority setting. Additionally, small scientific communities have refused to operate under such mechanisms arguing a transgression of the autonomy and independence of the scientific enterprise. The increasing relevance of research for economic development and the need to tackle global health problems have stimulated several initiatives to establish transparent and replicable priority setting exercises [34], [35], [36], [37]. The efforts have been directed to encourage the prioritization of research at country level to assure that research investments have a local impact. An increasing number of Latin American countries such as Brazil, Argentina, Peru and Cuba are implementing existing methodologies or generating new ones [38], [39]. In the case of Mexico, the creation of the CONACYT funds has led to an incipient environment of research priority setting. Considering the information available for FOSSIS, this fund represents a step forward to research planning, but the process requires some adjustments to accomplish the recently proposed criteria for health research prioritization (legitimacy, stakeholder involvement, revision and appealing, and leadership) [38], [40]. It is not clear how the general topics and subtopics are chosen to be included in the list of suitable projects to be supported. In the analyzed period, the call of proposals includes 20 topics that are divided in 139 subtopics. Thus, the prioritization exercise needs to be strengthened by assuring the participation not only of scientists but also of other groups in the society. Based on this analysis of the CONACYT funds, it can be concluded that the research portfolio in Mexico did not match the epidemiological transition of the country during the last decades. Health research is heavily oriented to infectious and parasitic diseases and environmental pollution effects, which are not the conditions of major impact according to the burden of disease measures. In contrast, the number of projects related to neuropsychiatric conditions, sense organ diseases, cardiovascular diseases, respiratory diseases, digestive diseases and injuries are underrepresented. This panorama suggests that besides better priority-setting practices it is also required to reform educational programs to focus research training on new health needs. Despite these weaknesses in priority-setting, the experience in the use of CONACYT funds indicates that research planning had a positive effect on the research profile. Fortunately, funding has started to be allocated on health problems in comparison to previous years [12]. Furthermore, the number of proposals of clinic research has increased as well as the proposals related with vaccine development, diagnostic methods and medical equipment.

Besides the identification of health research areas that require promotion, the intention of this work was to serve as an exercise of public accountability of one of the largest scientific communities in Mexico. The Mexican health research system has a long tradition that is reflected by solid research institutions and a growing community of human resources. The health research sector in Mexico has reached a size that requires continual evaluation. In this initial approximation, we found that funding allocation has a weaker association with burden of disease parameters than in other countries where similar analyses have been performed [16], [22], [31], [32]. To understand the cause of this problem, more comprehensive evaluations about the social impact of each health research area are required. Additionally, it will be necessary to open a debate about the convenience of using public funds to foster such a diverse research portfolio. As described here, the current panorama shows that health research budget is divided in small grants supporting a great diversity of topics which makes questionable the impact of these investments on the health problems, even in the long-term. This is an extremely important issue because science and technology have low levels of social and political recognition in low and middle income countries. Health sector offers a unique opportunity to change this vision. In Latin America, Mexico is the largest pharmaceutical market and is one of the OECD countries where drug expenditures reach the greatest share of all heath care public investment in the last decade [41], [42]. Development of a local pharmaceutical industry interacting efficiently with the health research system might be an alternative to reduce the cost of health care in the next years. The compass of pertinence for health research could be a way to aid improving health conditions of the population. In this respect, it is urgent to establish a clear and long lasting national research agenda.

## Supporting Information

Table S1 **CONACYT Research Funds and Measures of Disease Burden.**
(DOC)
