# Supplementary material for: Health Research Funding in Mexico: The Need for a Long-Term Agenda
Source: PLoS One. 2012 Dec 10;7(12):e51195. doi: 10.1371/journal.pone.0051195 (PMC3519634; doi:10.1371/journal.pone.0051195)
Supplement: Table S1 — CONACYT Research Funds and Measures of Disease Burden. (DOC) [file pone.0051195.s001.doc]

**Table S1.** CONACYT Research Funds and Measures of Disease Burden

| **Disease** |  | **Measure of Disease Burden^*^** | |
| --- | --- | --- | --- |
|  | **Funds** | thousands (rank) | |
|  | thousands of US$ PPP (%of total) | **DALY** | **Mortality** |
| Poisonings | 30,792,929 (9.28) | 29 (58) | 1 (43) |
| Diabetes mellitus | 24,144,698 (7.28) | 828 (3) | 52 (2) |
| Lower respiratory infections | 20,093,770 (6.06) | 365 (13) | 20 (9) |
| Infectious and parasitic diseases | 19,440,290 (5.86) | 147 (32) | 6 (20) |
| Endocrine disorders | 16,755,487 (5.05) | 303 (15) | 6 (19) |
| Malignant neoplasms | 15,554,329 (4.69) | 202 (24) | 12 (14) |
| Diarrhoeal diseases | 14,077,600 (4.24) | 238 (21) | 5 (24) |
| Tuberculosis | 13,059,977 (3.94) | 54 (51) | 3 (32) |
| Cervix uteri cancer | 11,494,334 (3.46) | 78 (45) | 6 (23) |
| Dengue | 10,879,711 (3.28) | 1 (74) | 0 (61) |
| Breast cancer | 10,606,754 (3.2) | 76 (46) | 5 (25) |
| HIV/AIDS | 10,050,103 (3.03) | 143 (34) | 5 (26) |
| Nephritis and nephrosis | 9,464,468 (2.85) | 167 (29) | 14 (11) |
| Neuropsychiatric conditions | 7,010,928 (2.11) | 555 (8) | 4 (28) |
| Alzheimer and other dementias | 6,440,636 (1.94) | 146 (33) | 1 (40) |
| Musculoskeletal diseases | 6,258,870 (1.89) | 152 (31) | 2 (36) |
| Perinatal conditions (h) | 6,043,762 (1.82) | 1059 (1) | 25 (6) |
| Other unintentional injuries | 5,761,343 (1.74) | 493 (9) | 9 (16) |
| Cardiovascular diseases | 5,664,986 (1.71) | 130 (35) | 13 (13) |
| Maternal conditions | 5,512,656 (1.66) | 215 (23) | 1 (44) |
| Chagas disease | 4,944,484 (1.49) | 53 (52) | 0 (62) |
| Leukaemia | 4,635,567 (1.4) | 102 (42) | 4 (27) |
| Hypertensive heart disease | 4,586,786 (1.38) | 89 (44) | 13 (12) |
| Nutritional deficiencies | 4,215,466 (1.27) | 274 (19) | 4 (30) |
| Ischaemic heart disease | 4,077,887 (1.23) | 555 (7) | 71 (1) |
| Parkinson disease | 3,467,200 (1.04) | 11 (70) | 1 (47) |
| Digestive diseases | 3,415,078 (1.03) | 426 (11) | 25 (5) |
| Rheumatoid arthritis | 3,372,770 (1.02) | 128 (36) | 1 (46) |
| Gastrointestinal cancer | 3,184,974 (0.96) | 128 (37) | 1 (42) |
| Leishmaniasis | 2,808,997 (0.85) | 1 (73) | 0 (68) |
| Epilepsy | 2,789,011 (0.84) | 176 (26) | 2 (37) |
| Drug use disorders | 2,606,421 (0.79) | 96 (43) | 0 (54) |
| Hepatitis B&C | 2,593,576 (0.78) | 21 (64) | 1 (49) |
| Prostate cancer | 2,310,891 (0.7) | 30 (57) | 6 (22) |
| Trachea, bronchus, lung cancers | 2,298,991 (0.69) | 65 (49) | 8 (18) |
| Violence | 1,947,151 (0.59) | 415 (12) | 10 (15) |
| Lymphomas, multiple myeloma | 1,919,439 (0.58) | 51 (53) | 4 (31) |
| Osteoarthritis | 1,864,996 (0.56) | 193 (25) | 0 (52) |
| Childhood-cluster diseases | 1,852,830 (0.56) | 21 (65) | 0 (57) |
| STDs excluding HIV | 1,844,520 (0.56) | 108 (41) | 0 (56) |
| Sense organ diseases | 1,735,548 (0.52) | 855 (2) | 0 (64) |

*Mortality and disability-adjusted life-years lost were obtained from the 2004 update of the World Health Organization’s Global Burden of Disease Project.

Table S1 (continued)

| **Disease** |  | **Measure of Disease Burden^*^** | |
| --- | --- | --- | --- |
|  | **Funds** | thousands (rank) | |
|  | thousands of US$ PPP (%of total) | **DALY** | **Mortality** |
| Unipolar depressive disorders | 1,569,401 (0.47) | 798 (4) | 0 (59) |
| Malaria | 1,423,118 (0.43) | 0 (76) | 0() |
| Cerebrovascular disease | 1,264,905 (0.38) | 277 (18) | 33 (3) |
| Cirrhosis of the liver | 1,220,868 (0.37) | 458 (10) | 28 (4) |
| Multiple sclerosis | 1,113,115 (0.34) | 25 (61) | 0 (53) |
| Melanoma and other skin cancers | 953,820 (0.29) | 12 (69) | 1 (38) |
| Respiratory diseases | 948,926 (0.29) | 163 (30) | 8 (17) |
| Congenital anomalies | 927,853 (0.28) | 737 (5) | 14 (10) |
| Chronic obstructive pulmonary disease | 921,815 (0.28) | 230 (22) | 21 (7) |
| Road traffic accidents | 921,473 (0.28) | 625 (6) | 21 (8) |
| Intestinal nematode infections | 772,377 (0.23) | 19 (66) | 0 (60) |
| Schizophrenia | 744,800 (0.22) | 286 (16) | 0 (55) |
| Skin diseases | 731,690 (0.22) | 67 (48) | 0 (51) |
| Asthma | 675,873 (0.2) | 339 (14) | 2 (33) |
| Self-inflicted injuries | 673,405 (0.2) | 111 (40) | 4 (29) |
| Panic disorder | 649,444 (0.2) | 116 (39) | 0 () |
| Liver cancer | 559,157 (0.17) | 49 (54) | 6 (21) |
| Alcohol use disorders | 497,499 (0.15) | 283 (17) | 1 (45) |
| Bipolar disorder | 486,007 (0.15) | 239 (20) | 0 (65) |
| Genitourinary diseases | 392,434 (0.12) | 28 (59) | 2 (34) |
| Obsessive-compulsive disorder | 346,263 (0.1) | 126 (38) | 0 () |
| Respiratory infections | 284,263 (0.09) | 25 (60) | 0 (63) |
| Post-traumatic stress disorder | 281,970 (0.08) | 48 (56) | 0 (67) |
| Ovary cancer | 279,899 (0.08) | 23 (62) | 2 (35) |
| Migraine | 249,117 (0.08) | 175 (27) | 0 () |
| Rheumatic heart disease | 240,106 (0.07) | 18 (67) | 1 (41) |
| Dental caries | 235,727 (0.07) | 167 (28) | 0 () |
| Insomnia (primary) | 191,065 (0.06) | 74 (47) | 0 () |
| Periodontal disease | 153,017 (0.05) | 5 (71) | 0 (69) |
| Oral conditions | 152,941 (0.05) | 63 (50) | 0 (58) |
| Leprosy | 143,427 (0.04) | 3 (72) | 0 (66) |
| Onchocerciasis | 100,281 (0.03) | 0 (75) | 0 () |
| Inflammatory heart diseases (k) | 65,459 (0.02) | 22 (63) | 1 (39) |
| Benign prostatic hypertrophy | 30,753 (0.01) | 48 (55) | 1 (48) |
| Upper respiratory infections | 24,940 (0.01) | 13 (68) | 0 (50) |

*Mortality and disability-adjusted life-years lost were obtained from the 2004 update of the World Health Organization’s Global Burden of Disease Project.
